# Structural Enablers of Rare Disease Treatment Coverage in Latin America and the Caribbean: Lessons from Emicizumab

**DOI:** 10.3390/jmahp14010013

**Published:** 2026-02-25

**Authors:** Daniela Sugg Herrera, Dino Sepúlveda Viveros, Moisés Russo Namias, Natalia Garrido

**Affiliations:** 1 Sugg y Asociados, Santiago 7500593, Chile; 2 Faculty of Economics and Business, Universidad de Chile, Santiago 8330015, Chile; 3 Instituto de Ciencias e Innovación en Medicina, Facultad de Medicina Clínica Alemana, Universidad del Desarrollo, Santiago 7610658, Chile; 4 Fundación Arturo López Pérez, Santiago 7500921, Chile

**Keywords:** health care coverage, emicizumab, health system structure, LATAM

## Abstract

We examine how structural characteristics of health systems in Latin America and the Caribbean (LAC) shape access to innovative therapies, using emicizumab for hemophilia A as a case study. Although the therapy is available in the region, access remains uneven and constrained by high costs and fragmented health system arrangements. Using a descriptive structural approach, we characterize the health system configurations associated with financial coverage of emicizumab across 16 LAC countries, representing more than 85% of the regional population. Regulatory approval timelines and coverage status were described, and principal component analysis (PCA) was applied to synthesize multiple indicators into a Global Characteristics Index capturing five core health system functions: resource generation, financing, service delivery, general governance, and therapy-specific governance. Coverage is defined as formal access with explicit financial protection provided by the health system. Substantial heterogeneity was observed across countries. Regulatory approval was often achieved relatively rapidly, but this did not consistently translate into timely or comprehensive coverage. Countries with stronger structural characteristics—particularly in resource generation, service delivery, and governance—tended to achieve broader and more sustained coverage, although institutional capacity alone was not sufficient in all cases. Our results emphasize the need to strengthen health governance and adopt specific policies for rare diseases in the region.

## 1. Introduction

Rare diseases (RD) are a persistent challenge for health systems, due to their high economic burden and the need to develop specialized capabilities for their comprehensive management [1]. In Latin America and the Caribbean (LAC), an estimated 40–50 million people have an RD, in a context marked by structural barriers that hinder equitable access to care [2]. These difficulties emerge from limited epidemiological data, shortage of trained personnel, insufficient infrastructure, and low prioritization of these pathologies in public policies [2], compounded by chronically insufficient health sector funding below international standards. As a result, limitations in access to care for RD impede many countries in the region from achieving the universal health coverage goals of the 2030 Agenda [3].

Among rare diseases, hereditary hemophilia is particularly well characterized [1]. The most frequent variant, hemophilia A, is a genetic disorder of blood clotting caused by a deficiency of factor VIII, an essential protein for coagulation. This condition is inherited through the X chromosome, so it predominantly affects males, although females can be carriers and, in rare cases, manifest the disease [4]. Severity varies from mild to severe depending on factor VIII levels [4]. Standard treatment is based on replacement therapy with factor VIII concentrate, administered either during bleeding episodes (on demand) or as part of a prophylactic regimen. Prophylaxis in hemophilia A is an important strategy to improve patients’ quality of life and long-term health outcomes, avoiding the serious complications that can result from frequent bleeding episodes [5,6]. A key clinical challenge of prophylaxis is the development of neutralizing antibodies against the administered factor, known as inhibitors, that compromise prophylaxis effectiveness.

The therapeutic landscape for hemophilia A has evolved substantially beyond traditional factor replacement. Treatment has progressed through successive generations: plasma-derived concentrates, standard recombinant factors, extended half-life products, and now innovative non-factor approaches [7].

Emicizumab, a bispecific antibody mimicking factor VIII function, has been characterized as a “game-changer” for its subcutaneous administration and efficacy in patients with or without inhibitors [8]. Additional non-factor agents, including concizumab, marstacimab, and fitusiran, are already approved by regulatory agencies or in advanced development state [9], while gene therapies have demonstrated durable factor expression for up to five years in clinical trials [10]. Despite these advances, approximately 70% of patients globally lack access to effective treatment [11]. This evolution presents both opportunities and challenges: while newer therapies may reduce long-term burden, their costs and evaluation requirements strain already constrained health systems.

Evidence from high-income settings illustrates the magnitude of this challenge. Cavazza et al. [12] documented annual per-patient costs for hemophilia ranging from €6660 in Bulgaria to €194,490 in Germany, with pharmacological treatment representing nearly 90% of direct healthcare costs. Similar access challenges have been documented in the Middle East and North Africa, where increasing prevalence coexists with persistent barriers to comprehensive care [13]. These comparisons underscore that even modest European benchmarks exceed per capita health expenditure in several LAC countries, and that access barriers represent a global phenomenon in middle-income regions.

Emerging evidence from LAC itself quantifies these burdens more precisely. The recently published CHESS LATAM study, which analyzed financial burden for Argentina, Brazil, Chile, and Colombia, estimated annual per-patient costs for severe hemophilia of up to US$277,000 adjusting for purchasing power, with factor replacement therapy accounting for up to 99% of direct costs [14]. Additionally, mean EQ-5D-5L among a selected sample of patients was 0.59 (for hemophilia A) and 0.46 (for hemophilia B), highlighting the loss of quality of life that patients are exposed to [14]. Moreover, the health gap attributable to resource constraints is illustrated by the São Paulo-Toronto comparison, which demonstrated that Brazilian children with hemophilia had Pettersson radiographic scores four times higher (indicating worse joint damage) and significantly lower activity levels than their Canadian counterparts with equivalent access to prophylaxis [15].

Despite the availability of new technologies for treating hemophilia, access in low- and middle-income countries (LMICs), and specifically in LAC, continues to be both limited and disparate. In many countries, these treatments are not incorporated into public health systems’ coverage or reimbursement lists, due in part to their high cost, which in some cases exceeds US$200,000 per patient per year [16], while in others it is possible to observe universal coverage of those treatments.

In LAC 52% of the estimated population with coagulation disorders are medically identified, according to 2023 data from the World Federation of Hemophilia (WFH) [17]. Of this group, 57% have hemophilia A; within this subpopulation, 43.2% present a severe form of the disease, 20.5% a moderate form, and 28% a mild form. Marked heterogeneity exists between LAC countries in terms of the proportion of the population diagnosed, treatment access, and the availability of factor VIII concentrates.

Emicizumab is the first biospecific humanized monoclonal antibody that mimics the cofactor function of factor VIII in the body, administered subcutaneously as a prophylactic therapy to prevent or reduce bleeding episodes in patients with hemophilia A with or without inhibitors [18,19]. The FDA approved emicizumab for patients with inhibitors in November 2017 [20] and extended approval to patients without inhibitors in October 2018 [21]. A recent systematic review confirmed robust evidence of efficacy in non-inhibitor patients, demonstrating significant reductions in annualized bleeding rates with favorable safety profiles and high patient satisfaction [22].

Emicizumab presents a therapeutic innovation both for its degree of efficacy, reaching a rate of zero annual bleeds, and for its subcutaneous administration, which improves adherence and quality of life [18,19]. It also reduces direct costs associated with factor use, bypass agents, hospitalizations, and bleeding complications [23,24,25,26,27,28,29]. This profile of high efficacy in a relatively common rare disease makes it an excellent candidate for understanding how health systems manage access to innovation and offers insight into their readiness to confront future waves of high-cost innovation in rare diseases in the region.

Although heterogeneity in treatment access for rare diseases has been recognized as a barrier to achieving Universal Health Coverage, there is little analytical and quantitative evidence on the factors influencing decisions within national healthcare systems. Precedents on other types of interventions, such as vaccination, have been carried out: for example, Arsenault et al. [30] identified political stability, gender equality, smaller territorial area, and external financing as key determinants of DTP3 vaccine coverage in countries supported by the Global Alliance for Vaccines and Immunization (GAVI). Likewise, Feigl et al. [31] showed that variables such as GDP per capita, education, and certain political and social factors, such as income inequality (GINI coefficient) and democratic governance, are associated with higher formal health coverage. Whilst these studies provide valuable evidence on coverage decision-making processes, the public-health nature of vaccination differs fundamentally from rare disease treatment. The specific challenges of rare diseases, including their invisibility within healthcare systems and their substantial economic and health burden, limit the transferability of such findings.

This study aims to identify and characterize country-level health system archetypes in LAC based on observed configurations of system attributes and treatment coverage. Coverage is defined as formal access to treatment with financial protection provided by the health system. Using emicizumab as a case study, the analysis adopts a descriptive, non-causal approach to examine how structural differences across health systems relate to patterns of access to therapies for low-prevalence diseases in the region.

## 2. Materials and Methods

We analyzed the effective coverage of emicizumab in 16 Latin American countries by classifying them according to the incorporation of hemophilia A treatment within their respective health schemes. This classification aims to characterize the level of financial protection provided by national insurance systems and to provide a standardized description of effective access across countries.

The central contribution of this study is a descriptive characterization of country-level health system attributes associated with differential coverage outcomes for a high-cost rare disease therapy. The analysis is explicitly framed from a health system capacity and supply-side perspective, focusing on the economic, institutional, and governance conditions that enable—or constrain—the adoption of innovative therapies.

Given the multidimensional nature of these attributes and the absence of comparable panel data, we employ principal component analysis (PCA) as a dimensionality-reduction and data-synthesis technique, not as a causal modeling strategy. PCA was used to construct a composite Global Characteristics Index, composed of sub-indices capturing five core health system functions: Health Service Provision, Resource Generation, Health Financing, General Governance, and Specific Governance. These functions are adapted from the WHO health system framework [32] to the specific context of this rare disease and therapy.

The resulting index allows countries to be positioned in a comparative analytical space according to their structural characteristics and observed coverage levels, facilitating the identification of country profiles and typologies. Importantly, this approach does not aim to estimate causal relationships between system attributes and coverage outcomes but rather provides a first empirical step toward systematically characterizing enabling conditions for access to innovative therapies in Latin America and the Caribbean.

### 2.1. Data Sources and Indicators

The countries included in the study were Argentina, Brazil, Bolivia, Chile, Colombia, Costa Rica, Cuba, Dominican Republic, Ecuador, El Salvador, Guatemala, Mexico, Panama, Paraguay, Peru, and Uruguay, together representing more than 85% of the population of the ECLAC region [33]. Venezuela was excluded due to the generalized lack of reliable and comparable data across key variables. Cuba was included only in the descriptive assessment of regulatory timelines and coverage but was excluded from the principal component analysis due to the absence of core structural data. To preserve a relevant and representative regional sample for the structural assessment, selective data imputation strategies were applied, recognizing that this approach involves trade-offs and limitations, which are explicitly discussed in the manuscript.

The analysis is based on a wide range of data sources, mainly from secondary and open access sources, such as the records from the WFH [34], World Bank (WB) [35], World Health Organization (WHO) [36,37], International Monetary Fund (IMF) [38] and World Intellectual Property Organization (WIPO) [39]. In addition, specific information on coverage and access to treatment with emicizumab was provided directly by the company that owns the technology used as a case study. Additional methodological details, including data sources and parameter values, are presented in the Supplementary Materials (Table S1).

In this study, the term access has two dimensions: (i) regulatory access, which refers to the physical availability and approval by the health authority of the treatment in the country, and (ii) financial access, which represents the decision of the health system to grant financial protection through formal mechanisms. We will use the term coverage to account for the percentage of the population that uses the treatment with formal financial protection.

Coverage is a dynamic phenomenon. To describe its evolution, two observation points were defined. The first corresponds to the initial moment in which financial access to emicizumab was granted in each country, which occurred close in time to the first FDA approval of the therapy and is referred to as the baseline period (*t* = 0). The second corresponds to the most recent observed situation, reflecting the current coverage status between 2023 and 2024, referred to as the current period (*t* = 1) (In the case of Mexico, information on the date of submission to the regulatory authority was not available; therefore, the time between submission and approval was estimated assuming that the FDA approval date coincided with the submission date.).

Country-level structural attributes were constructed using data organized into predefined time windows to ensure cross-country comparability and to capture medium-term structural characteristics rather than short-term fluctuations. For each indicator, a single reference window was selected and applied consistently across all countries. The primary reference window corresponded to the most recent period with sufficient regional coverage (2021–2024). When data were not available for this window for a given indicator, the immediately preceding window (2018–2020) was used for all countries, followed, if necessary, by an earlier period (2006–2017), and subsequently by 2000–2005.

This data imputation strategy was applied selectively with the objective of maintaining a relevant and comparable regional sample, while minimizing sensitivity to short-term fluctuations and isolated data gaps. The inclusion of information from different time windows does not imply a longitudinal analysis or temporal ordering of effects but rather supports the construction of stable composite indices that characterize the structural capacity and institutional context of health systems. Coverage measures were not included in the PCA or in any statistical model and are used exclusively for descriptive comparison and quadrant-based positioning.

In the specific case of the Dominican Republic, data were not available for the Universal Health Service Coverage (UHSC) indicator and the number of clinical studies. To preserve the inclusion of the country in the regional comparative analysis, values for these indicators were imputed using the average of a reference group of countries with broadly comparable structural characteristics. This reference group comprised Brazil, Colombia, and Mexico and was defined using a Euclidean distance–based clustering procedure applied to the set of general country-level indicators included in the analysis, excluding therapy-specific regulatory variables and the indicators subject to imputation. As these indicators enter the analysis as part of composite sub-indices that are subsequently integrated into a higher-level index, the potential influence of imputed values on the overall results is attenuated.

### 2.2. Financial Access to Emicizumab in the Region

Based on the information provided by the company, complemented by the analysis of data on the number of patients and financial protection systems in each country, a categorization of emicizumab financial access was established for the treatment schemes available for patients with and without inhibitors. Although the categorization of this study presents similarities with the categories used by FIFARMA [40] in its W.A.I.T. report, it adopts a more simplified approach and with stricter criteria regarding the type of financial protection considered. The categorization of financial access, as a variable of analysis, is as follows:

1. None: No patients have coverage through public or private insurance system. Also included in this category are cases of legal protection.
2. Limited: The study and reimbursement of patients on a case-by-case basis by public or private insurance system.
3. Partial: Public or private insurance systems generate protocols and define coverage by subpopulation based on specific criteria within the indication and patient profile approved by the regulatory agency.
4. Advanced: Public or private insurance systems establish expanded coverage, based on the potential population profile defined in the indication approved by the regulatory agency.

Country assignments to each category were based not only on the predefined framework but also on consistency with the number of patients effectively covered, as reported by the licensee company. The classification process was reviewed jointly by the authors to ensure internal consistency, and the resulting categorization was assessed for coherence with existing international benchmarks, including the W.A.I.T. framework, and with known country-specific coverage arrangements. In cases where reported patient numbers were inconsistent with the nominal coverage category, countries were reclassified accordingly, for example when explicit budgetary restrictions limited the practical application of coverage.

### 2.3. Outcome Variable: Coverage of Emicizumab

For countries classified as ‘with financial access,’ the specific level of coverage was estimated, while those without any type of provision were directly assigned zero coverage. The estimation was approached from two perspectives: one based on the identified hemophilia A population and the other on the total potential affected population. The number of identified patients corresponds to the number of people diagnosed and registered with a coagulation disorder, while the number of potential patients corresponds to the total number of people who could have a bleeding disorder, based on the known prevalence of the disease in the general population [17]. The first method reflects countries’ capacity to care for diagnosed patients, while the second provides a standardized and comparable measure of the extent of coverage, independent of diagnostic gaps. Since these gaps vary significantly between countries [17] and may bias the comparison, the main analysis is based on coverage for the total potential population.

For coverage over identified patients, data on the number of patients treated and the number of patients identified with hemophilia A were used. Specifically, for each country and each period “*t*”, this was estimated as:

(1) $$Coverage\;among\;{Identified}_{t}\;=\;\frac{\#\;of\;Patients\;Treated_{t}}{\#\;of\;Identified\;Hemophilia\;A\;Patients_{t}}$$

To estimate effective coverage over the potential population with hemophilia A, the number of patients treated was related to the estimated total potential population. The latter was obtained by adjusting the number of identified patients for country-specific diagnostic gaps. Specifically, the potential population was estimated as the number of identified patients divided by the proportion of patients identified in each country, as reported by the WFH [4]. This approach allows coverage estimates that are comparable across countries with different levels of case detection.

(2) $$Effective\;Cover{age}_{t}=\frac{{\#of\;Patients\;Treated}_{t}}{\frac{\#\;of\;Identified\;Hemophilia\;A\;Patients_{t}}{Identification\;Rate_{t}}}$$

For illustration, consider a country with 50 patients treated with emicizumab. If 200 patients with hemophilia A have been identified and the overall identification rate for bleeding disorders is 60%, the estimated potential population is 200/0.60 = 333 patients. Effective coverage over the potential population is therefore 50/333 = 15.0%.

### 2.4. Analytical Framework: Health System Structural Attributes

In the last two decades, multiple conceptual frameworks have been developed to analyze the functioning and performance of health systems [41,42,43,44]. Among them, the WHO Analytical Framework [32] stands out, which facilitates evaluating systems based on their essential functions: service provision, resource generation, financing, and governance. These functions form the operational basis of the system, and their analysis provides an analytical framework to assess and contextualize patterns of effectiveness, efficiency, and equity.

In this study, these four essential functions are considered along with a fifth—specific governance—to characterize the case of emicizumab. These dimensions are used to analyze their influence on treatment coverage, measured in relation to the potential population, combining quantitative and qualitative variables to assess the capacity and performance of the health system.

Table 1 details the variables used to characterize each health system function. The resource generation dimension is represented by three variables, as is the financing dimension, while service delivery includes two. General governance has four constituent variables and specific governance has two. For example, resource generation considers indicators such as the availability of beds and hospitals. Service delivery includes both the universal coverage index and case study-specific variables such as the use of factor VIII. General governance reflects aspects such as institutional stability and innovation development, while specific governance characterizes regulatory processes related to access to therapy through approval timelines for different indications. The inclusion of separate timelines for patients with and without inhibitors reflects distinct but sequential regulatory decisions for the same therapy, which together capture country-level regulatory capacity and efficiency in the context of emicizumab access.

### 2.5. Principal Component Method

A principal component analysis (PCA) was used to reduce the dimensionality of the variables within each function, and to subsequently synthesize the information from the five functions into an aggregate index to relate it to effective coverage. For each health system function, indicators were constructed using more than one recent observation when available, to improve measurement robustness and reduce sensitivity to isolated data gaps, reflecting the relatively stable structural nature of health system characteristics.

Then, an archetype was generated of countries according to their health system structure characteristics and their coverage results. This statistical method transforms a set of potentially correlated variables into a reduced number of uncorrelated elements, known as principal components, which capture most of the variability present in the data [45]. Each of these components represents a distinct linear combination of the variables included, which generates a factor loading scheme specific to each component. Before applying principal component analysis, each variable was standardized to have a mean of 0 and a standard deviation of 1, a standard preparatory procedure for PCA.

Hence, it is possible to calculate an index from each component, synthesizing the information from all the variables into a single value. Although indexes can be developed for each component, the first component generated is the one that explains the largest proportion of the total variance, making it the most effective summary of the general pattern of variation among the variables. In the present study this component is used as reference to synthesize the variables associated with each function into a single index.

Once the index has been calculated for each function, principal component analysis (PCA) is again performed on the five indices obtained, which are now considered as sub-indexes. This allows the construction of a global indicator that integrates the information of all the functions, called the Global Characteristics Index (GCI).

Finally, a quadrant ranking analysis is performed to compare the GCI with the outcome variable: the effective coverage of emicizumab over the potentially treatable population. To do this, the average of both variables is calculated and reference lines are drawn on the graph, dividing the plane into four quadrants: (i) upper left quadrant: countries with low GCI but high coverage; (ii) upper right quadrant: countries with high GCI and high coverage; (iii) lower left quadrant: countries with low GCI and low coverage; (iv) lower right quadrant: countries with high GCI but low coverage.

This graphic approach makes it possible to visualize the relationship between the structure of a health system and the country with health coverage, facilitating the identification of patterns and differences between countries, the construction of national archetypes, and the formulation of differentiated health policy recommendations.

## 3. Results

### 3.1. Regulatory Timing of the Therapy

**Figure 1:**
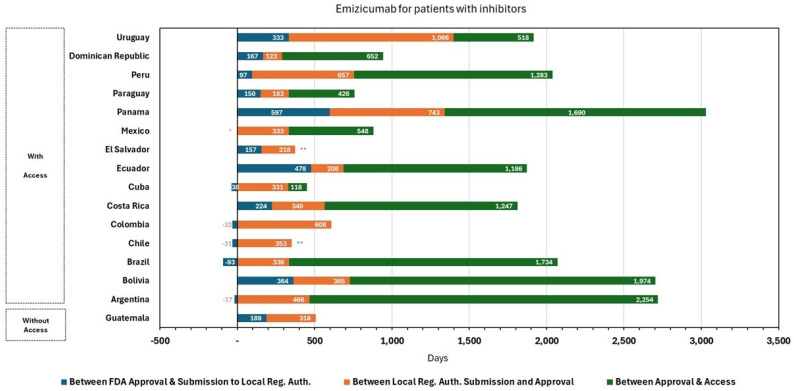
Emicizumab for Patients with Inhibitors: Approval and Access Durations Across Countries. Note: An asterisk (*) indicates that no official regulatory approval date was available, while double asterisk (**) indicates that no official access date was available. In these cases, dates and durations are estimated based on the best available country-level information as of the time of the study, including regulatory status and reported access conditions, even if formal access had not yet been established. Source: Author elaboration.

**Figure 2:**
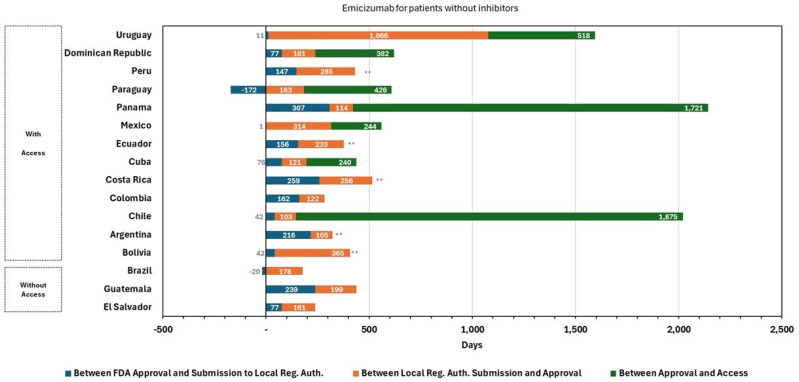
Emicizumab for Patients without Inhibitors: Approval and Access Durations Across Countries. Note: A double asterisk (**) indicates that no official access date was available. In these cases, dates and durations are estimated based on the best available country-level information as of the time of the study, including regulatory status and reported access conditions, even if formal access had not yet been established. Source: Author elaboration.

In the case of emicizumab, regulatory approvals were granted first for the indication in patients with inhibitors (Figure 1), with an average evaluation time of 416 days (<12 months) from submission. The Dominican Republic presented the shortest regulatory time with 123 days, while the longest was Uruguay with 1066 days. Regarding financial access, the average time elapsed between sanitary authorization and access was 1048 days. However, in Colombia, access was granted together with sanitary authorization. On the other hand, Argentina took the longest time to obtain access, approximately six years. Considering the entire process, i.e., the elapsed time between filing of the application and access, the average time for all countries was four years. Subsequently, the indication was approved for patients without inhibitors (Figure 2) within an average evaluation time of 247 days (<9 months), with a minimum of 103 days from Chile and a maximum observed in Uruguay with 1066 days. To grant financial access, the systems took an average of 676 days, except for Colombia, which granted regulatory approval and financial access together, and Brazil took 2222 days to grant financial protection.

These differences highlight substantial heterogeneity in regulatory processes across countries, which may reflect differences in institutional arrangements, regulatory capacity, and reliance on external reference mechanisms.

### 3.2. Financial Access

For patients with inhibitors there was limited financial access in 25% (*n* = 4) of cases and advanced financial access in 31% (*n* = 5) at *t* = 0. Coverage was focused and initiated mainly in pediatric populations. In a period of less than 3 to 4 years, significant progress was made in the health systems, achieving the expansion of partial and advanced protection for both pediatric and adult patients. Evidence of this is that in *t* = 63% (*n* = 10) have advanced access, and only Guatemala remains without financial access (Table 2).

The situation differs for patients without inhibitors, where in *t* = 1 only 13% of the cases (*n* = 2) have advanced access (Panama and Colombia), and the focus of access was once again for pediatric patients. Progress in coverage has allowed partial access in 50% of the cases (*n* = 8), which has required the establishment of protocols and criteria for patient inclusion and exclusion. During the analyzed period, the countries of El Salvador, Guatemala and Brazil do not present formal coverage for this indication in *t* = 1 (Table 3).

The slower pace of coverage expansion observed in some indications, particularly among patients without inhibitors, reflects the structural frictions faced by health systems when incorporating high-cost innovative therapies. Although these treatments offer substantial improvements in prophylaxis and quality of life, their universalization remains constrained by affordability and budgetary pressures. Consequently, access has been implemented gradually, with a clear prioritization of pediatric patients and the use of partial coverage schemes supported by restrictive protocols, underscoring the central role of financial and governance constraints in shaping real-world access.

### 3.3. Population Coverage

**Figure 3:**
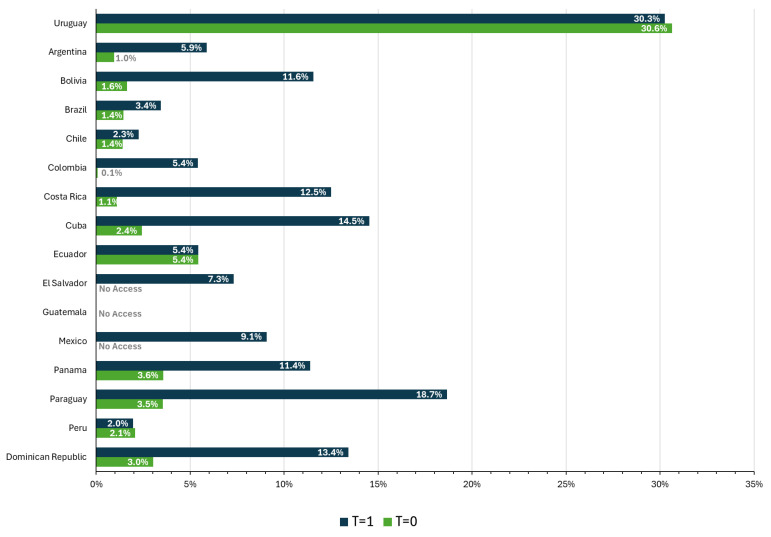
Financial coverage for the identified population, baseline and follow-up. Source: Author elaboration.

**Figure 4:**
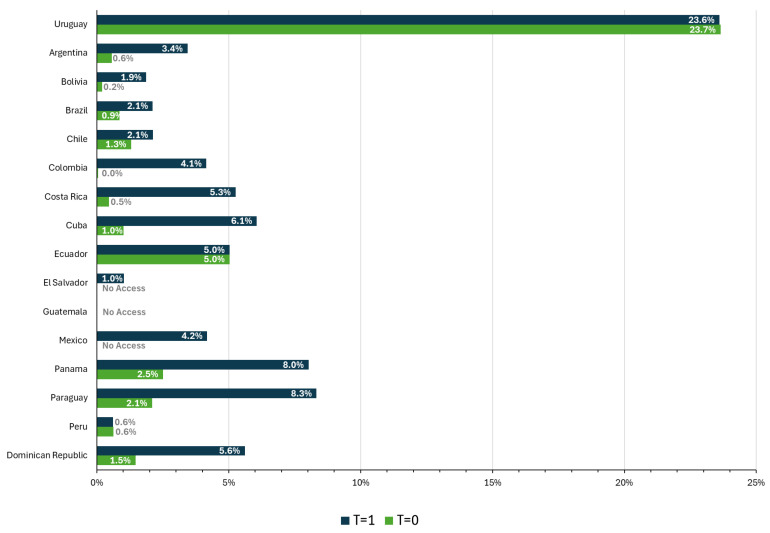
Financial coverage relative to the potential population, baseline and follow-up. Source: Author elaboration.

Figure 3 and Figure 4 report coverage, i.e., the percentage of the population using therapy with financial access, according to the identified population (Equation (1)) and the potential population (Equation (2)), respectively, for *t* = 0 and *t* = 1. Both estimates show the variability in the region, as well as how many countries have a population identification gap for this health problem, which generates the significant difference observed between the overall levels of coverage.

There was clear progress in coverage between the first and second evaluation periods. On average, coverage increased from 2% of the population (identified or potential) to 8% of the identified population and 6% of the potential population. The largest increase in coverage was in Paraguay, with an increase of 15 percentage points (p.p.), followed by Cuba, with an increase of 12 p.p. However, in both countries the increase in coverage is twice as high for the identified population as for the potential population. Chile also recorded relatively low progress, with only 0.8 and 0.9 p.p. for the identified and potential populations, respectively.

In both observation periods, Uruguay had the highest coverage and Peru the lowest, both for the identified and potential population. In the last period, Peru only covered 0.6% of the potentially affected population, while Uruguay reached about 24%.

Beyond country-specific differences, both figures reveal a systematic gap between coverage estimates based on identified versus potential populations, suggesting that limitations in case identification remain a major determinant of observed coverage levels in the region.

### 3.4. The Global Characteristics Index: A Measure of Enabling Factors for Coverage

Table 4 shows the results of the model that quantifies the factor loadings of each variable in each component. In all indexes, the variables have positive factor loadings, which implies that higher values raise the index score. The only exception is the Gini coefficient, whose negative weighting reflects its nature as an indicator of inequality: higher values reduce the governance index. As all variables are positively ordered, a higher index value indicates a better performance of the country in that dimension.

The Resource Generation Index primarily reflects installed health system capacity, as it is driven by hospital beds and physicians per capita, while clinical studies play a smaller, secondary role related to research capacity rather than innovation per se.

In the Financing Index, public and government health expenditure as a share of GDP show the highest factor loadings, indicating that fiscal effort is the main driver of this dimension. GDP per capita contributes positively but with a lower weight, suggesting that financing capacity reflects policy choices more than income levels alone.

The Service Provision Index reflects both overall system capacity and disease-specific service delivery, being driven by universal service coverage and the use of Factor VIII per capita, which captures the existing level of care for patients with hemophilia A.

The General Governance Index is mainly driven by innovation and institutional quality indicators. The Global Innovation Index and the Institutions Index present the highest factor loadings, highlighting the relevance of broader governance and innovation environments. The Gini coefficient exhibits a negative factor loading, reflecting its role as an inequality indicator, whereby higher inequality levels reduce overall governance performance within this component.

Finally, the Specific Governance Index is driven by the time between submission and approval for treatments with and without inhibitors. The identical factor loadings indicate a consistent regulatory responsiveness across both indications.

Regarding the GCI, all sub-indices display positive factor loadings, indicating that overall system performance reflects the combined contribution of multiple health system functions. The Service Delivery Index shows the highest factor loading, followed closely by the Resource Generation Index, suggesting that operational capacity and effective service provision are the main dimensions differentiating countries. In contrast, the lower contribution of the Specific Governance Index indicates that regulatory timing exhibits lower relative variability across countries compared to other system dimensions. Table 5 presents the average of each sub-index and the Global Characteristics Index (GCI) for each country, providing a comprehensive comparison of health systems. The main results are detailed below.

1. Resource Generation Index: Argentina achieves the highest score with a value of 3.9, followed by Uruguay and Panama in third position. In contrast, Guatemala registers the lowest value with −2.4.
2. Financing Index: The best-ranked countries are Argentina and Uruguay, with Brazil close behind. On the other hand, Guatemala and the Dominican Republic have the lowest scores in this index.
3. Service Delivery Index: Chile leads this dimension with a score of 2.4, followed by Argentina, Uruguay and Colombia. Once again, Guatemala obtains the lowest value (−3.6), accompanied by Bolivia with −2.9.
4. General Governance: Chile ranks first, followed by Mexico and Uruguay. At the other extreme, Bolivia, Guatemala and Ecuador have the lowest scores.
5. Specific Governance Index: Uruguay leads this index with a score of 4.4, followed by Peru. On the other hand, Panama, the Dominican Republic and Paraguay have the lowest values.

Beyond individual rankings, Table 5 reveals distinct health system profiles across countries. Uruguay and Argentina show consistently high performance across most dimensions, which is reflected in their leading GCI scores. In contrast, countries such as Chile and Brazil display strong performance in specific dimensions—service delivery and governance in the case of Chile, and financing in the case of Brazil—while presenting more moderate results in others. At the lower end, Guatemala, Bolivia and Paraguay consistently rank poorly across multiple sub-indices, indicating persistent structural limitations rather than isolated weaknesses. Overall, the GCI appears to reflect balanced system performance rather than leadership in a single dimension. As a robustness exercise, the GCI was recalculated excluding the Specific Governance Index related to hemophilia-specific regulatory processes. The results show that the exclusion of this dimension leads to minimal changes in the GCI values for most countries, indicating that overall cross-country variation in the composite index remains largely driven by differences in service delivery, resource generation, and financing capacities. A notable exception is Uruguay, where the index decreases from 3.43 to 2.62, while the relative position of the remaining countries remains largely unchanged. The full results of this counterfactual analysis are reported in the Supplementary Materials (Table S2).

### 3.5. Health System Functions and Emicizumab Coverage

**Figure 5:**
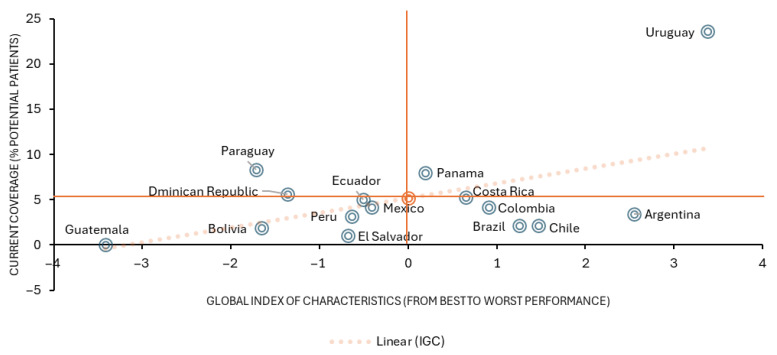
Relationship between Country Characteristic Set and Current Hemophilia Treatment Coverage. Note: The reference lines (orange) indicate the midpoints of the variables and divide the figure into four quadrants to facilitate descriptive interpretation. The dotted reference line corresponds to a linear fit included for visualization purposes only and does not represent a statistical or inferential model. Source: Author elaboration.

Figure 5 presents a descriptive visualization of the joint distribution between the Global Characteristics Index (GCI) and the current level of coverage, measured as the percentage of patients treated relative to the potentially affected population. Countries with higher GCI values tend to be positioned toward higher levels of coverage, illustrating a consistent pattern across the sample. The reference line included in the figure serves solely as a visual aid to facilitate interpretation of this pattern and does not represent an inferential or predictive model. Based on this descriptive representation, countries can be grouped into four quadrants:

1. Quadrant 1 (upper left-low GCI and high coverage): Paraguay and Dominican Republic;
2. Quadrant 2 (upper right—with high GCI and high coverage): Uruguay, Panama and, at the margin, Costa Rica;
3. Quadrant 3 (lower left—low GCI and low coverage): Guatemala, Bolivia, Ecuador, Peru, El Salvador and Mexico;
4. Quadrant 4 (lower right—high GCI and low coverage): Chile, Argentina, Brazil and Colombia.

Countries located in Quadrant 2 are distinguished by their high levels of governance, both general and specific. A representative example is Uruguay, where the National Resources Fund (FNR), created in 1980, finances highly complex medical procedures and, since 2005, also high-cost drugs. This structure guarantees broader and sustained access to innovative therapies.

In contrast, Quadrant 3 countries lack robust financial protection programs for high-cost diseases. According to Mayrides et al. [31], although some countries have enacted specific laws on rare diseases and there are timely initiatives, effective implementation still has notable shortcomings.

Thus, a country with a more robust set of characteristics in terms of the essential functions of a health system (GCI), and especially with strengths in Resource Generation and Specific Governance, as is the case of Uruguay, shows greater linkage and better results in access to innovative therapies, as the emicizumab case illustrates.

## 4. Discussion

Rare diseases pose a sustained challenge for health systems, particularly in Latin America and the Caribbean (LAC), where structurally constrained public financing limits the capacity to absorb high-cost, long-term innovations. Public health expenditure in the region remains below 6% of GDP on average [3], with a substantial share of health spending borne through out-of-pocket payments, in contrast to European systems that allocate between 6% and 9% of GDP through solidarity-based financing mechanisms and reach total health expenditure levels of approximately 9.2% [46]. These differences are highly relevant for rare diseases, whose management requires sustained financing, specialized services, and long-term institutional commitment. While evidence from high-income settings shows that conditions such as hemophilia impose substantial economic and quality-of-life burdens even in well-resourced systems, the challenge in LAC is compounded by tighter fiscal space, weaker information systems, and greater uncertainty in coverage decision-making. In this context, emicizumab—although disease-specific and relatively recent—serves as an informative tracer of how health systems in the region respond to advanced, high-cost therapies for rare diseases. Patterns of access to emicizumab therefore reflect not only clinical or regulatory considerations, but broader structural and institutional capacities, offering insight into the readiness of LAC health systems to manage current and future waves of innovation in rare disease care.

This study adopts a descriptive and structural analytical approach to examine health system attributes associated with effective financial coverage of emicizumab across 16 LAC countries. Principal component analysis was used to synthesize multiple indicators into a Global Characteristics Index reflecting five essential health system functions: resource generation, financing, service delivery, general governance, and therapy-specific governance. This approach does not aim to establish causal relationships, but rather to characterize enabling conditions and identify system archetypes relevant for policy design in a context where comparable longitudinal data are scarce.

By focusing on the joint configuration of health system attributes rather than on individual determinants, the archetype-based approach adopted in this study provides a complementary perspective to conventional explanatory analyses. This framework allows for the identification of recurrent system configurations that shape access outcomes under conditions of structural constraint and data scarcity. In the context of small cross-country samples and limited longitudinal information, such as in LAC, this approach avoids overinterpretation of marginal effects and instead supports differentiated policy reasoning tailored to distinct system profiles.

Several findings have direct policy relevance. First, regulatory approval processes for emicizumab in the region were, on average, relatively agile compared with regional benchmarks for innovative medicines, such as those reported in the FIFARMA W.A.I.T. survey. However, this regulatory responsiveness did not translate into timely access with financial protection. A substantial gap persists between regulatory authorization and effective coverage, delaying treatment availability and limiting population-level health gains. This regulatory–financial disconnect suggests that the principal barriers to access lie not in regulatory capacity per se, but in financing mechanisms and governance arrangements.

The heterogeneity observed in regulatory approval timelines across countries requires cautious interpretation. Differences in time to approval may reflect variation in institutional capacity, regulatory pathways, the use of reliance or reference mechanisms, and national prioritization of rare disease therapies, rather than differences in regulatory efficiency alone. Accordingly, regulatory timelines are interpreted as descriptive indicators of governance arrangements within broader health system archetypes, rather than as direct measures of regulatory performance. Further comparative research examining regulatory frameworks would be required to assess regulatory quality and efficiency more systematically across countries. Second, coverage expansion followed a clear pattern of prioritization. Access was introduced earlier and more consistently for patients with inhibitors—often beginning with pediatric populations—while coverage for patients without inhibitors expanded more slowly and frequently under restrictive protocols. This reflects both clinical considerations, probable public pressure specific to coverage of children and fiscal constraints, illustrating how affordability pressures shape access pathways even for therapies with strong clinical value.

Beyond the observed patterns of prioritization, the findings highlight important limitations in how coverage for high-cost rare disease therapies is defined and evaluated in practice. In several countries, coverage is formally granted but remains constrained by restrictive eligibility criteria, budget caps, or limited implementation capacity, resulting in a gap between de jure coverage and effective access. This underscores that coverage should not be assessed solely in binary terms, but rather along a continuum that considers the breadth of eligible populations, the degree of financial protection, and the consistency of access over time. In the context of rare diseases, where patient numbers are small but per-patient costs are high, such distinctions are particularly relevant for understanding the real-world impact of coverage decisions.

It is important to note that this study adopts a deliberate and restrictive definition of coverage, focused on formal access to treatment with explicit financial protection provided by the health system. Under this framework, access through judicialization, off-label use, or other informal mechanisms is not classified as coverage, even though such pathways may enable treatment at the individual level. This distinction reflects the analytical focus of the study on institutionalized financing arrangements and system-level capacity, rather than on ad hoc or case-based access mechanisms.

Third, countries with stronger structural characteristics, particularly in service delivery, resource generation, and governance, tend to achieve broader and more sustained coverage. Conversely, countries with low coverage typically exhibited cumulative weaknesses across multiple system functions, rather than isolated deficiencies. Notably, several countries with relatively strong structural capacity nonetheless displayed limited coverage, indicating that institutional capacity is necessary but not sufficient. In these cases, factors such as benefit package design, budget fragmentation, prioritization rules, and political economy dynamics likely play a decisive role.

The quadrant analysis highlights that a substantial subset of countries remains trapped in an unfavorable equilibrium characterized by both low systemic capacity and low effective coverage. For these systems, achieving meaningful improvements is unlikely to be feasible through marginal reforms alone. Strengthening governance, improving information systems, and expanding fiscal space are necessary but challenging objectives, particularly in a context of modest regional economic growth. IMF [47] projections suggest average growth of approximately 2.5% for LAC in the coming years, compared with a global average of 3.3%, further constraining the resources available for health system expansion.

Although emicizumab represents a specific therapeutic innovation, its role in this analysis extends beyond hemophilia A. As a high-cost, long-term therapy requiring sustained financing and coordinated governance, emicizumab functions as an early stress test of health system readiness for advanced innovation in rare diseases. The challenges observed in achieving timely and effective financial coverage for emicizumab anticipate even greater difficulties associated with emerging gene therapies and other potentially curative interventions, which entail higher upfront costs, irreversible budget commitments, and heightened uncertainty. In this sense, patterns of access to emicizumab provide forward-looking insight into how health systems in the region may confront future waves of innovation.

Taken together, the findings offer actionable policy insights. Countries with low capacity and low coverage may benefit most from external technical assistance, regional cooperation, and the development of explicit rare disease financing mechanisms. For countries with stronger system attributes but delayed access, reforms should focus on benefit design, budget integration, and transparent prioritization frameworks for high-cost therapies. Given the small patient populations involved, regional approaches to health technology assessment, procurement, and evidence generation may offer viable pathways to improve access while preserving financial sustainability.

Building on these findings, the analytical framework developed in this study points to several directions for future research. In particular, country-level case studies could use the archetypes identified here as an entry point to examine how specific institutional arrangements, benefit design choices, and financing strategies shape coverage decisions in different national contexts. In addition, assessing the causal impact of policy levers—such as the introduction of dedicated financing mechanisms or changes in regulatory pathways—would require longitudinal data and study designs explicitly oriented toward causal inference. The present study provides a comparative and structural foundation to guide such subsequent analyses, rather than attempting to address these questions directly.

This study has several limitations that should be considered when interpreting the findings. Coverage estimates rely in part on manufacturer-provided data, which may not fully capture all channels of treatment use and may be subject to measurement error, despite representing the most standardized and comparable source available for a multicountry analysis. In addition, the categorization of financial access, while based on explicit criteria and internal consistency checks, was not externally validated. Finally, the analysis does not incorporate qualitative evidence on national decision-making processes or the role of patient advocacy, nor does it distinguish between formal coverage and effective access at the patient level. These limitations underscore the need for more comprehensive data systems and mixed-methods approaches in future research.

## 5. Conclusions

Increasing financial resources alone is insufficient to ensure access to treatments for rare diseases; institutional reforms are required to reduce fragmentation, improve coordination between regulatory and financing processes, and establish explicit benefit entitlements. The absence of such mechanisms not only delays access but also fuels judicialization as a compensatory and often inequitable access pathway, rather than as a sustainable policy solution.

Addressing rare diseases therefore requires moving beyond prevalence-based criteria alone toward public policies that incorporate considerations of equity, economic burden, social impact, and management efficiency. Strengthening governance for decision-making—through transparent and timely health technology evaluations, clearer prioritization rules, and well-defined procedures for incorporation into coverage schemes—remains central to this effort. Importantly, these challenges do not affect all countries equally, but vary according to distinct health system configurations, as reflected in the archetypes identified in this study.

In this context, Latin America and the Caribbean face a complex scenario. Improving financial coverage for rare diseases requires reinforcing governance frameworks and redefining political and budgetary priorities within constrained fiscal environments. Although this analysis focuses on hemophilia A and the case of emicizumab, the findings are intended to inform broader approaches to other high-cost, low-prevalence therapies entering health systems in the region.

Our results contribute to a more comprehensive understanding of how structural and policy factors shape access to innovation in rare diseases. From a policy perspective, the findings underscore the need to strengthen institutional priority-setting arrangements and sustainable financing mechanisms capable of narrowing the persistent gap between regulatory approval and effective patient access. This imperative will become increasingly salient as more transformative and high-cost innovations reach the market.

## Figures and Tables

**Table 1:** Classification of variables to be used in groups of essential functions.

Essential Function		Variables
(1) Resource Generation		Number of Doctors (per 1000 population)
		Number of Hospital Beds (per 1000 population)
		Number of Clinical Studies (per 1000 inhabitants)
(2) Financing		Gross Domestic Product Per Capita (at constant prices, PPP)
		Government Expenditure (% GDP)
		Public Health Expenditure (% GDP)
(3) Service Delivery		Use of Factor VIII Per Capita
		Universal Health Service Coverage Index (UHSC)
(4) Governance	(a) General	Gini Coefficient
		Research & Development Index
		Institutions Index
		Global Innovation Index
	(b) Specific	Days between Submission of Hemophilia Inhibitor Treatment to Local Regulatory Authority and Approval
		Days between Submission of Treatment Without Hemophilia Inhibitors to Local Regulatory Authority and Approval

Notes: Data for Group (1) were obtained from the World Bank data repository and the World Health Organization International Clinical Trials Registry Platform (ICTRP). Data for Group (2) were sourced from the World Bank. Data for Group (3) were obtained from the World Federation of Hemophilia and the World Health Organization. Data for Group (4a) were sourced from the World Bank and the World Intellectual Property Organization. Data for Group (4b) were derived from internal pharmaceutical company data. Source: Author elaboration.

**Table 2:** Coverage categorization of treatment with inhibitors.

Category	*t* = 0		*t* = 1		Change	
	*n*	%	*n*	%	*n*	*p.p.*
None	4	25%	1	6%	−3	−18.8
Limited	4	25%	1	6%	−3	−18.8
Partial	3	19%	4	25%	1	6.3
Advanced	5	31%	10	63%	5	31.3
Total	16	100%	16	100%		

Source: Author elaboration.

**Table 3:** Coverage categorization of treatment without inhibitors.

Category	*t* = 0		*t* = 1		Change	
	*n*	%	*n*	%	*n*	*p.p.*
None	6	38%	3	19%	−3	−18.8
Limited	8	50%	3	19%	−5	−31.3
Partial	1	6%	8	50%	7	43.8
Advanced	1	6%	2	13%	1	6.3
Total	16	100%	16	100%		

Source: Author elaboration.

**Table 4:** Composition of the PCA Resulting Component by Group of Variables.

(1)	(2)	(3)	(4)
Group	Variable	Time	Factor Loads
Resource Generation	Number of Physicians	*t* = −1	0.56
	Number of Hospital Beds	*t* = −1	0.53
	Number of Hospital Beds	*t* = 0	0.57
	Number of Clinical Studies	*t* = 1	0.21
	Number of Clinical Studies	*t* = 0	0.20
Financing	GDP Per Capita	*t* = 0	0.19
	GDP Per Capita	*t* = 1	0.18
	Government Spending (% GDP)	*t* = 0	0.46
	Government Expenditure (% GDP)	*t* = 1	0.45
	Public Health Expenditure (% GDP)	*t* = 0	0.52
	Public Health Expenditure (% GDP)	*t* = 1	0.49
Service Provision	Use of Factor VIII Per Capita	*t* = −1	0.48
	Use of Factor VIII Per Capita	*t* = 0	0.47
	Universal Health Service Coverage Index	*t* = 0	0.52
	Universal Health Service Coverage Index	*t* = 1	0.53
Overall Governance	Gini Coefficient	*t* = −2	−0.20
	Gini Coefficient	*t* = −1	−0.05
	Research & Development Index	*t* = 0	0.31
	Research & Development Index	*t* = 1	0.34
	Global Innovation Index	*t* = 0	0.46
	Global Innovation Index	*t* = 1	0.45
	Institutions Index	*t* = 0	0.42
	Institutions Index	*t* = 1	0.40
Specific Governance	Date between Submission and Approval with Inhibitors	-	0.71
	Date between Submission and Approval without Inhibitors	-	0.71
Global Characteristics Index	Resource Generation Index	-	0.50
	Financing Index	-	0.46
	Service Ratio	-	0.51
	General Governance Index	-	0.44
	Specific Governance Index	-	0.30

Note: The time variable corresponds to grouped observation periods used in the PCA estimation. Specifically, *t* = −2 refers to the period 2000–2005; *t* = −1 to 2006–2017; *t* = 0 to 2018–2020; and *t* = 1 to 2021–2024. Source: Author elaboration.

**Table 5:** Descriptive Statistics for Essential Function Indices.

	(1)	(2)	(3)	(4)	(5)	(6)
Country	Resource Generation	Financing	Service Delivery	Governance		General Characteristics Index
				General	Specific	
Argentina	3.87	2.57	1.71	0.94	−0.20	2.52
Bolivia	−1.27	0.93	−2.89	−3.56	0.28	−1.64
Brazil	0.56	1.31	1.34	2.01	−0.36	1.24
Chile	0.28	0.72	2.42	3.00	−0.54	1.46
Colombia	−0.41	1.30	1.54	0.92	0.26	0.90
Costa Rica	0.02	−0.15	1.43	1.60	−0.12	0.68
Ecuador	−0.66	1.01	0.16	−2.33	−0.61	−0.51
El Salvador	−0.56	0.43	−0.29	−1.70	−0.75	−0.66
Guatemala	−2.36	−3.75	−3.59	−2.86	−0.35	−3.41
Mexico	−1.06	−1.53	−0.62	2.35	0.03	−0.42
Panama	0.86	0.24	0.64	−0.24	−1.10	0.22
Paraguay	−1.02	−1.40	−1.53	−1.92	−0.79	−1.69
Peru	−0.73	−1.43	−1.35	0.73	0.89	−0.64
Dominican Republic	−0.59	−2.34	−0.65	−1.24	−1.03	−1.47
Uruguay	3.06	2.10	1.68	2.29	4.40	3.43
Average	0.00	0.00	0.00	0.00	0.00	0.00
Max.	3.87	2.57	2.42	3.00	4.40	3.43
Min.	−2.36	−3.75	−3.59	−3.56	−1.10	−3.41

Notes: The indexes presented in columns (1)–(5) are the result of performing a principal component analysis (PCA) on the variables belonging to each group, detailed in Table 1. The index in column (6) is the result of performing a PCA on the sub-indices of columns (1)–(5). The table presents the predicted score for each country using the first component yielded by the PCA. Source: Author elaboration.

## Data Availability

The data presented in this study are available on request from the corresponding author due to confidentiality and legal restrictions related to non-public coverage information provided by a third party.

## Supplementary Materials

The following supporting information can be downloaded at: https://www.mdpi.com/article/10.3390/jmahp14010013/s1, Table S1: Data sources used in the analysis of health system characteristics and emicizumab coverage; Table S2: Global Characteristics Index (GCI): baseline and counterfactual without Specific Governance.

## Abbreviations

The following abbreviations are used in this manuscript:

LAC	Latin America and the Caribbean
PCA	Principal Component Analysis
GCI	Global Characteristics Index
RD	Rare Diseases
GNP	Gross Domestic Product
GAVI	Global Alliance for Vaccines and Immunization
DTP3	Diphtheria, Tetanus, and Pertussis (3rd dose)
LMIC	Low- and middle-income Countries (LMICs)
WFH	World Federation of Hemophilia
FVIII	Factor VIII
FDA	Food and Drug Administration
ITI	Immune Tolerance Induction
APC	Activated Prothrombin Complex
ECLAC	Economic Commission for Latin America and the Caribbean
WB	World Bank
WHO	World Health Organization
WIPO	World Intellectual Property Organization
W.A.I.T.	Waiting to Access Innovative Therapies
p.p.	percentage points
OECD	Organisation for Economic Co-operation and Development
PAHO	Pan American Health Organization
IMF	International Monetary Fund
