# Supplementary material for: Structural Enablers of Rare Disease Treatment Coverage in Latin America and the Caribbean: Lessons from Emicizumab
Source: J Mark Access Health Policy. 2026 Feb 25;14(1):13. doi: 10.3390/jmahp14010013 (PMC13027683; doi:10.3390/jmahp14010013)
Supplement: Supplementary file 1 [file jmahp-14-00013-s001.zip › jmahp-4055802-supplementary.pdf]

Supplementary Materials

Table S1. Data sources used in the analysis of health system characteristics and emicizumab coverage

**Data Source (Organization)**	**Website Link**	**Indicators used**	**Type of data**	**Scope of data**
World Federation Hemophilia (WFH))	https://wfh.org/research-and-data-collection/annual-global-survey/	Identified people with hemophilia A, B, and unkown UI_FVIIIpercapita	Registry-based, self-reported by countries	Country-level, aggregate national
World Health Organization (WHO)	https://www.who.int/clinical-trials-registry-platform https://www.who.int/data/gho/indicator-metadata-registry/imr-details/4834	N° clinical Trials UHC SCI Population	Administrative and registry-based	Country-level, aggregate nationa
World Bank (WB)	Wbopendata: https://datahelpdesk.worldbank.org/knowledgebase/articles/889464-wbopendata-stata-module-to-access-world-bank-data	Physicians per 1,000 inhabitants Hospital beds per 1,000 people Public Health Expenditure % of GDP Gini Index GDP percapita (PPP)	Administrative and registry-based	Country-level, aggregate nationa
World Intellectual Property Organization (WIPO)	https://www.wipo.int/en/web/global-innovation-index	Global Innovation Index: Global Innovation Index	Composite index (secondary data)	Country-level, aggregate national
International Monetary Fund (IMF)	https://www.imf.org/external/datamapper/datasets	GDP growth Government debt (% of GDP) Government expenditure (% of GDP)	Administrative and registry-based	Country-level, aggregate national
Pharmaceutical company	-	Coverage Emicizumab Regulatory date of submitted Regulatory date of approval	Company-reported administrative data	Country-level, aggregate national

Note: All data sources correspond to secondary data from publicly available databases, except for coverage estimates and regulatory timelines for emicizumab, which were provided directly by the technology manufacturer. These data were used exclusively for descriptive purposes and were not all included in the PCA estimation.

Source: Author elaboration.

Table S2. Global Characteristics Index (GCI): baseline and counterfactual without Specific Governance

Country	(1)<br>GCI<br>(Baseline)	(2)<br>GCI<br>(Excluding Specific Governance)	(3)<br>(2)-(1)<br>Difference
Argentina	2.52	2.60	+0.08
Bolivia	−1.64	−1.77	−0.13
Brazil	1.24	1.33	+0.09
Chile	1.46	1.66	+0.20
Colombia	0.90	0.87	−0.03
Costa Rica	0.68	0.80	+0.12
Ecuador	−0.51	−0.39	+0.12
El Salvador	−0.66	−0.48	+0.18
Guatemala	−3.41	−3.48	−0.07
Mexico	−0.42	−0.46	−0.04
Panama	0.22	0.55	+0.33
Paraguay	−1.69	−1.55	+0.14
Peru	−0.64	−0.90	−0.26
Dominican Republic	−1.47	−1.41	+0.06
Uruguay	3.43	2.62	**−0.81**

Source: Author elaboration.
